# A historical perspective on the neurobiology of speech and language: from the 19th century to the present

**DOI:** 10.3389/fpsyg.2024.1420133

**Published:** 2024-09-18

**Authors:** Pascale Tremblay, Simona M. Brambati

**Affiliations:** ^1^École des Sciences de la Réadaptation, Université Laval, Québec City, QC, Canada; ^2^Centre de Recherche CERVO, Québec City, QC, Canada; ^3^Département de Psychologie, Université de Montréal, Montréal, QC, Canada; ^4^Centre de Recherche de l’Institut Universitaire de Gériatrie de Montréal, Montréal, QC, Canada

**Keywords:** speech, language, models, history, brain stimulation, brain imaging (CT and MRI), EEG

## Abstract

In this essay, we review 19th century conceptions on the neurobiology of speech and language, including the pioneer work of Franz Gall, Jean-Baptiste Bouillaud, Simon Alexandre Ernest Aubertin, Marc Dax, Paul Broca, and Carl Wernicke. We examine how these early investigations, anchored in the study of neurological disorders, have broadened their scope via neuropsychological and psycholinguistic theories and models. Then, we discuss how major technological advances have led to an important paradigm shift, through which the study of the brain slowly detached from the study of disease to become the study of individuals of all ages, with or without brain pathology or language disorders. The profusion of neuroimaging studies that were conducted in the past four decades, inquiring into various aspects of language have complemented—and often challenged—classical views on language production. Our understanding of the “motor speech center,” for instance, has been entirely transformed. The notion of cerebral dominance has also been revisited. We end this paper by discussing the challenges and controversies of 21st century neurobiology of speech and language as well as modern views of the neural architecture supporting speech and language functions.

## 1 Introduction

The purpose of this article is to trace the origins of the neurobiology of speech and language in the nineteenth century and explore its methodological and conceptual evolution alongside emerging disciplines like neuropsychology, neurolinguistics, and brain imaging. What began as an inquiry into neurological disorders has evolved into a vibrant, multidisciplinary field that combines basic and applied research. Technological advancements have catalyzed a paradigm shift, expanding research from pathology-focused investigations to research encompassing individuals of all ages, with or without brain or language disorders. Over the past four decades, a proliferation of neurophysiological and neuroimaging studies has both complemented and challenged traditional perspectives on speech and language. Finally, we address contemporary challenges, controversies, and future research directions in 21st-century neurobiology of speech and language.

## 2 Early work

**Figure 1:**
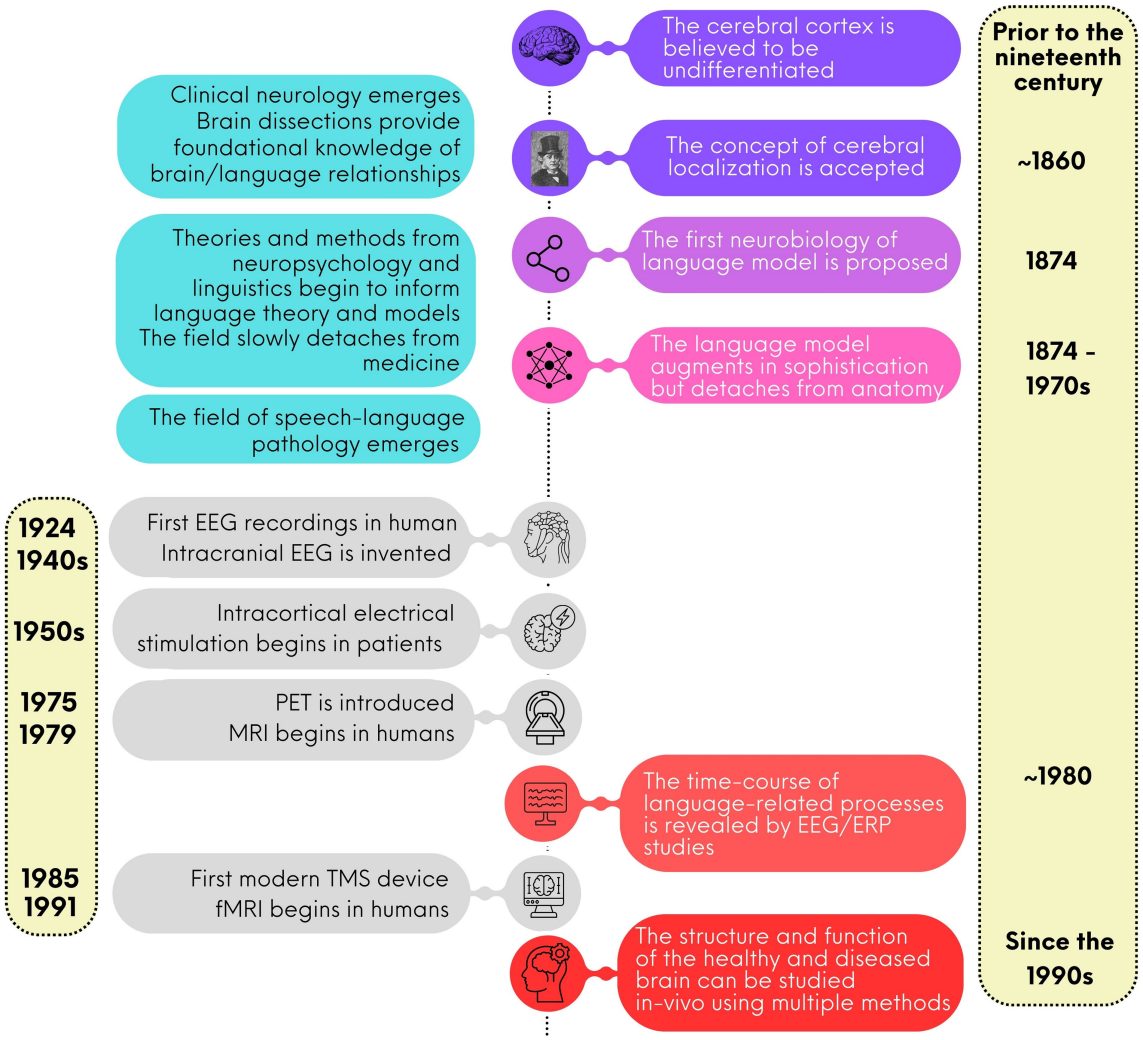
A brief timeline for the study of language neurobiology.

Up until the end of the 18th century, scientists believed that the human cerebral cortex was an undifferentiated organ that participated in all functions, from solving mathematical equations to talking and walking (see Figure 1 for a schematized timeline). One of the first scientists to question this notion was the German physician and anatomist Franz Joseph Gall (1758–1828). Gall’s fundamental idea was that each part of the brain had a specialization. For instance, he believed that the “language function” was localized in the anterior frontal cortex, behind the eyes. Gall also believed that the anatomy of the skull reflected the underlying brain organization. While the first notion was correct, the second was not. Yet *all* of Gall’s ideas were attacked and rejected by its contemporary scientists, particularly Marie-Jean-Pierre Flourens (1794–1867), an accomplished French physician (Yildirim and Sarikcioglu, 2007) Gall is largely remembered for his work on craniometry, which was renamed *phrenology* (and slightly revised) by Johann Spurzheim (1776–1832), who had worked with Gall for many years. Despite the controversies surrounding *phrenology*, it is important to acknowledge that his work prompted the scientific community to consider the idea that the brain is not a single, undifferentiated organ (Finger, 2000) a notion that is now widely accepted.

Gradually, during the 19th century in Europe and beyond, clinical neurology research began to flourish. Medical doctors turned to investigations of patients with neurological conditions, documenting their symptoms and conducting dissection after their death to understand the organization of the cerebral cortex. Jean-Baptiste Bouillaud (1796–1881) was a medical doctor at the Charité Hospital in Paris. Bouillaud was an ardent advocate of cerebral localization. He studied hundreds of clinical cases of people with brain damage to understand the functions of the different part of the cerebral cortex. In 1825, Bouillaud presented a paper at the Royal Academy of Medicine in Paris supporting a relationship between speech and the frontal lobe. In 1826 and again in 1830, he argued for the existence of two speech centers, one in the anterior frontal cortex, either left or right (Stookey, 1963), and one in the posterior part of the second frontal convolution of the left hemisphere. During the same period, Marc Dax (1770–1837), a small-town doctor from the south of France, had collected records of approximately 40 cases of acute speech defects from the literature and another 40 cases from his own medical practice. By 1836, Dax became convinced that the left hemisphere played a special role in speech production. While there is a possibility that Dax presented his findings in 1836 in Montpellier; he died before he could publish them. His son, Gustave Dax (1815–1893), later fought for his father’s discovery to be recognized (Finger and Roe, 1996).

Back in Paris, the claims of Jean-Baptiste Bouillaud did not receive widespread approval. Forty years later, in April 1861, Simon Alexandre Ernest Aubertin (1825–1895), Bouillaud’s son-in-law, and a medical doctor at the Charité Hospital, presented a case at a scientific meeting of the Société d’Anthropologie. The case involved a patient whose brain was exposed due to a firearm injury. Aubertin described how applying pressure to the anterior lobe led to a temporary arrest of speech. He argued that only severe lesion to the anterior lobe would lead to speech disorders, addressing critics who cited cases of anterior lobe lesions without speech difficulties. However, Aubertin’s arguments, like those of Bouillaud and Dax, were largely ignored.

Only days after Aubertin’s presentation, the respected French physician and surgeon Paul Broca (1824–1880), founder of the Société d’Anthropologie, received a patient in his service, a man named Mr. Leborgne, who was unable to utter any other word than “tan.” After his death, a few days later, the autopsy revealed a lesion in the third frontal convolution. These findings were immediately presented by Broca at the Société d’Anthropologie in 1861 and received with enthusiasm. Broca called the inability to speak *aphemia*, meaning “without speech.” A few months later, Broca described another patient, Mr. Lelong, who was incapable of speaking any other words than yes, no, and always. Again, the autopsy revealed a lesion in the anterior part of the brain [however, almost 150 years later, a reanalysis revealed that the lesions of both patients extended beyond the third frontal convolution (Dronkers et al., 2007)]. Both patients exhibited severe production deficits but retained intact comprehension and intelligence. Broca was the first to attribute speech functions to a specific part of the brain, the inferior frontal gyrus, known at the time as the third frontal convolution. He studied several similar cases, reinforcing his conviction that language is localized and that the cortex consists of differentiated regions with specializations. Recognizing that the third frontal is near the motor cortex, Broca’s idea was that this region was involved in converting word representations into an articulatory code for production. In 1865, after examining several cases of speech loss associated with left hemisphere damage, Broca (1865) published his seminal paper on the special role of the left hemisphere for language (cerebral dominance). He concluded that the left hemisphere is dominant and responsible for language, indicating that the two hemispheres are not identical. Thus, two key concepts in modern neuroscience—cerebral localization and cerebral dominance—became prominent scientific discussions (Finger, 2000).

## 3 The first model

Broca pursued a long and prolific scientific career in neurology but he did not write about speech after 1877 (Finger, 2000) and he never wrote about how the faculty of articulate language connected with the rest of the brain. In 1874, several years after Broca’s seminal paper, the German physician Carl Wernicke (1848–1905) proposed a second language center, located in the temporal lobe, which he believed received information from the acoustic nerve, contained speech sound images and was integral to language comprehension (Wernicke, 1874/1977). Wernicke, like Broca, employed clinical neurology as heuristic. The patients he studied were able to speak but did not comprehend language although their intelligence was intact. Although this type of disorder had been long known, it had never been associated with a specific cortical site. Importantly, in the same publication, relying on the differential clinical presentations of the two aphasia types (motor and sensory), Wernicke derived the first general theory of the neural bases of language. In this model, two regions, “Broca’s area” (as it was named by Wernicke) and a temporal site (often referred to as Wernicke’s area), are connected through a *fibra propriae* (in modern terminology, the arcuate fasciculus). In this model, Broca’s area is depicted as a region involved in articulate language, while Wernicke’s area is associated with comprehension. Despite this model, Wernicke explicitly stated that Broca’s area cannot be the sole speech center, emphasizing the insula as well. Wernicke described Broca’s area as being part of the primary motor cortex in the first frontal convolution, forming a broad region that connects with motor nuclei in the medulla allowing it to control facial muscles and speech.

With this first model, the neurobiology of language was born. Because language disorders could occur without impairment of other intellectual abilities, aphasia was the first demonstration that selective brain damage could affect one class of learned behavior while sparing other classes, providing a strong case in favor of the localization of functions in the cerebral cortex.

## 4 How the first model evolved through neuropsychological and psycholinguistic research

Building on the foundational work of pioneers like Paul Broca and Carl Wernicke, early understandings of mind-brain relationships was derived from clinical studies of brain-damaged patients. This approach achieved two objectives. First, clinical observations helped identify which language subcomponent, such as repetition, comprehension and reading, could be selectively disrupted in brain-damaged patients. Second, post-mortem investigations contributed to understanding the relationship between linguistic deficits and the specific sites of brain damage. These discoveries significantly contributed to the evolution and redefinition of the theoretical models of normal language functioning, initially developed by Wernicke (1874/1977).

In 1885, the german physician Ludwig Lichtheim (1845–1928) published a connectionist model of language stemming from the work of Broca and Wernicke, often referred to as the Wernicke-Lichtheim model (Lichtheim, 1885). The model was inspired by the observation of new clinical presentations of aphasia that could not be explained by Wernicke’s model. The model was illustrated as a diagram and included: (1) the “A” auditory perception pole, corresponding to the Wernicke area, linked to the pathway for auditory information; (2) the “M” motor pole, corresponding to the so-called Broca’s area, linked to the motor output pathway, and (3) the “B” (from the German word for concept, *Begriff*) concept center, not related to a specific brain region. These centers were interconnected through various pathways. This model had two main objectives. The first was to understand the brain/behavior relationship in language. The second was to explain the main clinical profiles reported in aphasia patients in terms of damage to the main centers and pathways represented in the model. Lichtheim’s model, which was later revived and further elaborated by Geschwind (1970), marks a shift to a model that is focused on the connections among different “centres” of language rather than on the anatomical correspondence between language “centers” and their neuroanatomical correlates. It is also the origin of the classical categorization of different types of aphasia (e.g., Broca’s, Wernicke’s, conduction, global, transcortical) based on observed patterns of language impairment. Lichtheim’s model was later criticized for oversimplifying the complexity of aphasia and for neglecting the cognitive and functional aspects of language processing. The model’s categorical approach does not account for the variability and overlap of symptoms in aphasia. Further, the model emphasized lesion localization, while modern research shows that aphasia can result from damage to various brain networks and not just isolated regions.

Key discoveries based on single case studies were mainly made between the late 1800s and the early 1900s. However, by the mid-1900s, the neuropsychological community began to question this approach, noting its limited generalizability and inherent biases (selection and experimentation). This prompted a shift towards patient-group research methodologies, moving away from reliance on single cases. Despite this shift, group studies faced significant criticism during the 1980s. The main criticism was that group studies violated the homogeneity assumption for group studies, which is a prerequisite for valid statistical analyses. Group studies presuppose that the members of a group share common characteristics. However, even when grouped based on classical syndromes, patients with aphasia can present highly heterogeneous symptomatology or present similar symptoms with different underlying causes. As a result, this approach has been criticized for its multiple methodological flaws (Caramazza, 1984). While it is important to recognize this controversy, it is crucial to acknowledge that neither single-case nor patient-group studies are without limitations.

In summary, early aphasia studies were crucial in establishing the neurobiology of language and provided foundational knowledge still relevant today. However, these early models were simplistic (for a review of these limitations, see Dick and Tremblay, 2012; Tremblay and Dick, 2016) and based on the assumption that pathological cases can reveal healthy brain function, a notion now challenged. Additionally, post-mortem approaches had limited lesion accuracy and the time between clinical observation and post-mortem examination hindered brain/behavior analyses. At that time, methods for investigating healthy brain function or *in-vivo* anatomical characterization were unavailable, and animal models offered limited insights due to their less complex communication systems. The advent of electrophysiological and brain imaging techniques was a critical breakthrough, providing new ways to study brain mechanisms underlying language and enabling the development of more sophisticated neurobiological models.

## 5 From clinical and behavioral neurology to the cognitive neuroscience of language

This section explores how 20th-century technological advances shifted the study of language neurobiology from a medical endeavor focusing on brain disease to an interdisciplinary field examining individuals of all ages, with or without language disorders. We first review electrophysiological and brain imaging approaches and conclude with brain stimulation methods and their impact on understanding language neurobiology.

### 5.1 Electrophysiological approaches

The first surface (scalp) electrophysiological (EEG) recordings in humans was conducted in 1924 by a German psychiatrist named Hans Berger. Berger recorded the brain electrical activity of a young patient who underwent trepanation to remove a cervical tumor. Berger was the first to describe the relationship between mental activity and variations in the electrical signal. Berger (1929) published the results of his observations. In the century since, EEG has become an indispensable clinical and research tool.

**Figure 2:**
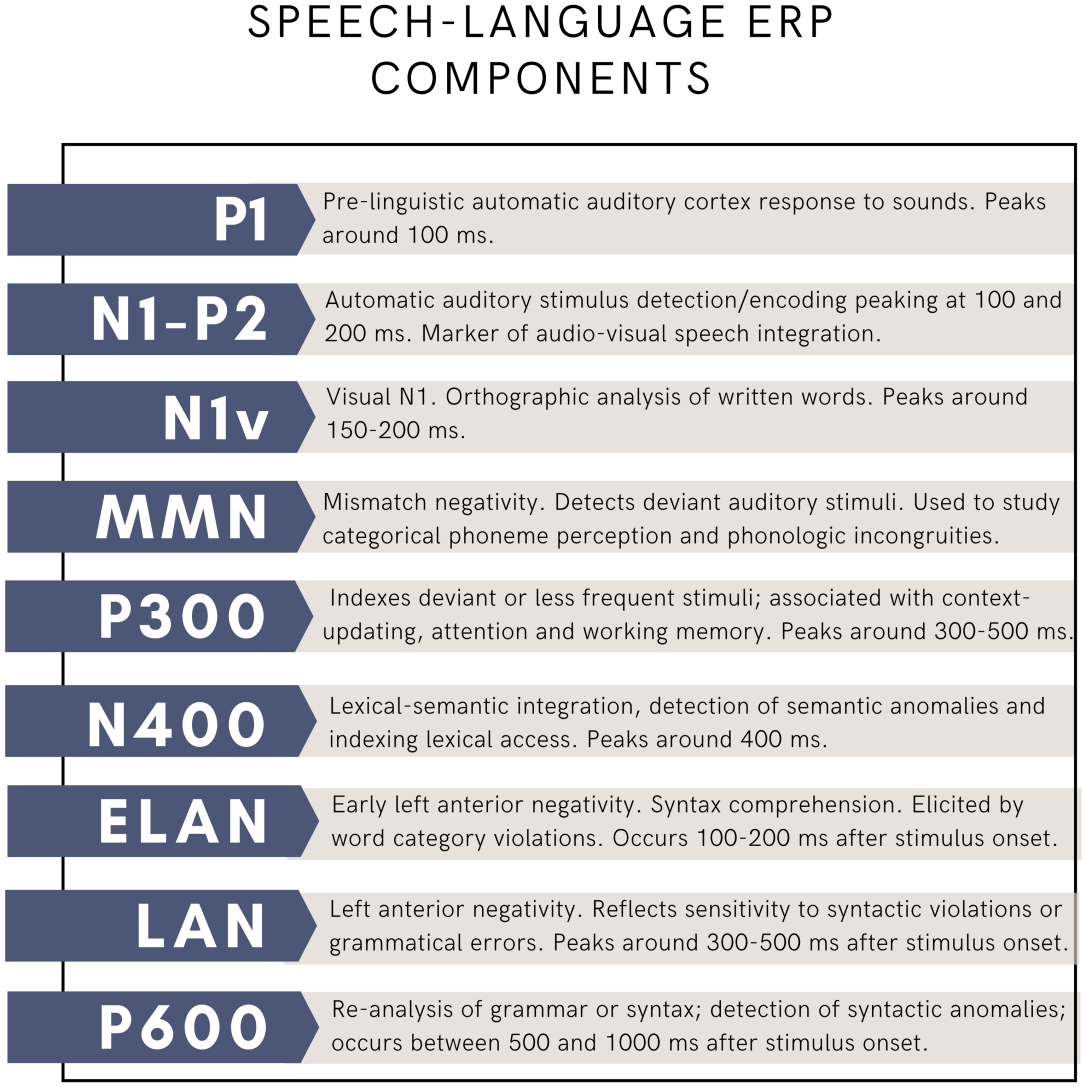
Main language-related ERP components.

The use of EEG in language research has had a tremendous impact on our understanding of underlying mechanisms. It allowed researchers to investigate how language processing unfolds in real time, and to monitor covert processing in the absence of an overt verbal response (for instance when listening to a story). Importantly, it also allowed researchers to test models of cognitive processes. To study language, scientists have relied on event-related brain potentials (ERP) since the 1980s (for thorough reviews of this topic, see Osterhout et al., 1997; Steinhauer and Connolly, 2008). ERPs are changes in electrical activity, which are evoked in response to repeated stimuli, such as viewing words on a computer screen or hearing spoken syllables. ERPs are obtained by averaging brain activity over multiple, often hundreds of trials. ERPs allow researchers to analyze the brain response to a particular stimulus, such as a syllable, a written or a spoken word, or even a sentence. The study of ERPs has offered very important insights in the study of language, revealing the precise timeline of brain responses to various speech and language processing mechanisms (Figure 2), and offering a window into how these processes evolve from childhood to older ages, and how they may be disrupted in disease, such as aphasia. While there exist other techniques to study the neurophysiological response to language, such as time-frequency analyses, and event-related synchronization and desynchronization, the ERP approach has dominated the field for decades. Among many discoveries, ERP studies have revealed the time course of the speech signal from the brainstem to the auditory cortex, and into more specialized phonological, lexical and syntactic processes. The N400 potential was the first language component that was characterized, which revealed that about 400 ms following the beginning of a sentence, detection of semantic anomalies can be measured, providing invaluable information about the time necessary to assess word meaning. More recent approaches include the study of brain signals at rest (resting-state EEG) as well as the study of continuous speech using speech tracking methods.

Alongside the development of surface-EEG, intracranial EEG was introduced in medicine for the diagnosis and treatment of intractable forms of epilepsy, Parkinsonism and other neurological disorders in the late 1940s and early 1950s (e.g., Jasper and Penfield, 1949). This method, with submillimeter and millisecond precision, involves placing electrodes directly on the surface of the brain to record electrical activity from awake patients. While less widespread because of its invasiveness, intracranial recording techniques have been instrumental in understanding brain oscillations, that is, that different frequency bands (e.g., gamma, beta, theta) are associated with different aspects of cognition and language processing (for a review, see Lachaux et al., 2012). For instance, gamma-band activity is often linked with language tasks that involve complex processing and integration. In clinical settings, electrocorticography (ECoG) is used for pre-surgical mapping in patients undergoing epilepsy surgery (Roessler et al., 2019). It helps identify critical language areas to avoid during surgery, thereby improving surgical outcomes and preserving language functions (Roessler et al., 2019).

### 5.2 Brain imaging approaches

Electrophysiological approaches have greatly advanced our understanding of language processing, but the advent of functional neuroimaging methods with higher spatial resolution in the 1990s marked a breakthrough for the neurobiology of language. Key techniques include positron emission tomography (PET, 1975) and functional magnetic resonance imaging (fMRI, 1991). Both methods map brain regions with increased blood flow linked to neural activity, but fMRI, due to its lower cost, versatility, and reduced invasiveness, has become more widely used than PET.

In the early years of functional imaging, researchers commonly used blood oxygenation level-dependent (BOLD) fMRI to study brain activity during language tasks. BOLD fMRI detects changes in blood oxygen levels, with increased neural activity leading to higher oxygen demand. As active neurons require more oxygen, blood vessels dilate, enhancing blood flow. fMRI measures these changes by detecting differences in magnetization between oxyhemoglobin and deoxyhemoglobin, providing an indirect measure of neural activity. The use of fMRI in the field of neurobiology of language did not replace clinical studies, but instead provided a complementary approach for studying functional language networks. In the early years following the introduction of these techniques, language neurobiology research primarily focused on individuals with post-stroke aphasia (PWA) or healthy controls. Research on PWA mainly aimed to investigate task-based fMRI activation pattern in brain-damaged patients with acquired language disorders (Fischer-Baum and Campana, 2017). The most common approaches were: (1) the patient(s)/controls comparison and (2) the pre−/post-treatment comparison. The first approach was primarily used to determine the differences between the activation pattern of PWA compared to controls engaged in a speech or a language task. The second approach aimed to assess whether aphasia treatments can promote brain reorganization in individuals with aphasia (PWA). This approach encompassed both single-case and group studies, though it faced limitations related to the generalizability of findings and group homogeneity. In this context, neuroplasticity referred to the functional reorganization that occurs during aphasia recovery. Although the definition of neuroplasticity is not unanimous and has generated debate over the years, these studies have contributed to promoting the notion that the brain of PWA is capable of reorganization and remodeling and that such reorganization can mediate speech/language recovery.

While functional MRI has become popular for studying language, morphological investigations aimed at characterizing brain damage/behavioral associations in clinical populations including PWA have also provided much needed information. The development of new methods for studying brain morphological characteristics based on *in-vivo* brain images provided new tools to overcome methodological limitations of post-mortem studies and, consequently, gave a new impulse to this line of research. One of the most frequently cited limitations of post-mortem studies was its limited anatomical precision. The use of high-definition anatomical MR images has allowed better *in-vivo* characterization of the stroke-induced brain damage and more precise definition of brain lesions. This led to more accurate investigation of the relationship between language and lesion data in case studies and for the emergence of larger-scale group studies testing the generalizability of language/lesion relationship findings based on single-case reports. Lesion approach in patient group studies generally aims at mapping brain regions that are commonly damaged in patients presenting a specific speech/language deficit. In this optic, patients presenting a specific symptom are grouped together, the contour or the lesion is reconstructed for each patient and a common area of injury is calculated. In some studies, the common area of injury is compared to a group of patients who do not present the language deficit of interest (Rorden and Karnath, 2004). Introduced in the early 2000s, voxel-based lesion-symptom mapping (VLSM) emerged as an alternative to traditional lesion analysis approach (Bates et al., 2003). Unlike methods that group patients based on clinical profiles, VLSM allows for the inclusion of patients with diverse language profiles and widely distributed lesions. This technique uses behavioral scores and lesion data at the voxel level as continuous variables. VLSM analyses determine, for each voxel, the extent to which damage in that area disrupts the specific language function under investigation. According to the authors, “*VLSM is an improvement on the previous lesion—symptom mapping techniques because it uses all available information, eliminating reliance on cutoff scores, clinical diagnoses or specified regions of interest*” (Bates et al., 2003). One of the main limitations of this approach is that it has limited sensitivity in detecting stroke-induced damages along white matter fibre bundles. In 2017, the connectome-based lesion-symptom mapping (CLSM) was proposed as a complementary method for studying brain/behavior relationship in lesioned patients in terms of white matter connections (Gleichgerrcht et al., 2017). This approach leverages on principles of VSLM combined to structural connectome (whole-brain structural connectivity reconstruction) providing a method for network assessments that goes beyond the site of gray matter lesion’s location. CLSM is thus a powerful approach to study the neurobiological basis of behavior.

While post-stroke aphasia remains a primary focus in the field of language neurobiology, research on primary progressive aphasia (PPA) has significantly advanced our understanding of brain damage and language symptoms. PPA, characterized by progressive loss of verbal communication abilities resulting from degeneration of language networks, was first described by Picks in 1892, though the modern definition of PPA was introduced by Mesulam (1982, 1987). Research in the early 2000s revealed the heterogeneity of PPA (Gorno-Tempini et al., 2004). In 2011, an international panel of experts introduced a common framework that classifies primary progressive aphasia (PPA) into three main variants, each defined by specific diagnostic criteria (Gorno-Tempini et al., 2011). Each variant is defined in terms of a cluster of language deficits and unique distribution of cortical atrophy. Over the years, these variants have provided a unique model for studying brain damage/language deficit relationship. Most of the original MRI studies on PPA focused on the correlation between the severity of language symptoms and gray matter atrophy, with the aim to map the brain regions where local atrophy was associated with the emergence and progressive deterioration of specific language functions. With the increasingly accepted view that neurodegenerative diseases do not target specific and isolated brain regions, but rather large-scale brain networks, the study of PPA, like the study of PWA, shifted from a localizationist approach to a network-based approach in which damage to white matter structural connections plays a central role (Seeley et al., 2009), leading to an increase in the number of neuroimaging studies focusing on white matter fibre bundles. By measuring the diffusion of water molecules in biological tissues, diffusion MRI (dMRI) allows the reconstruction of white matter tracts and provides information about the underlying microstructural properties of white matter (Jones, 2010), including myelination, fiber orientation, axon diameter and axon density. Nowadays, the study of PPA, as well as the study of all language dysfunctions, relies on the entire set of state-of-the-art MRI methods, including task-based and resting state functional MRI studies as well as structural and diffusion studies. While task-based fMRI studies measure brain activity by detecting changes in blood flow while participants perform specific tasks, such as language tasks, resting-state fMRI (rs-fMRI) studies measure brain activity while participants are not engaged in any explicit task, typically resting quietly. This method can reveal spontaneous brain activity and functional connectivity between brain regions, providing insights into the brain’s intrinsic network organization and functional architecture.

Alongside their application in studying PWA, PPA, and other speech and language disorders, the advent of brain imaging tools in the 1990s also opened a new research avenue focused on healthy participants. At first, most studies focused on young adults (mostly university students), but eventually the field became more diversified to include older adults and children. In the early days, studies on healthy participants relied massively on task-based fMRI to identify brain regions that are functionally involved in specific language tasks. In the first years, it was not uncommon for studies to be designed to test hypotheses derived from patients’ observations. For instance, case studies on patients reporting a double dissociation between noun and verb processing, or living and nonliving item naming, encouraged the development of fMRI activation studies aimed at mapping whether the processing of nouns and verbs or living and nonliving implicated different or overlapping brain networks. But this line of research also developed away from patient research to ask questions focused on understanding the basic building blocks of speech and language such as the neural representation of syllables, phonemes and words, the comparison of auditory and speech processing, the similarities between speaking and other complex motor actions, the organization of phonological working memory, the processing of syntax and semantics and much more (for a review, see Price, 2010). These studies revealed that the language network is extensive, encompassing regions that serve both specific language-related functions and broader domain-general functions.

The full set of MRI methods is now used to inquire after multiple aspects of human communication. Structural MRI studies have been used to examine not only the impact of development and aging on brain structure (including volume as well as cortical thickness) and communication, but also the neural basis for inter-individual differences in speech-language skills in the healthy brain, including reading, speech processing in noise and many others (for a review, see Richardson and Price, 2009). Diffusion MRI has enabled the detailed dissection and anatomical characterization of fiber bundles, leading to the identification of new pathways that are important for speech and language, beyond those traditionally associated with the arcuate fasciculus, including the uncinate fasciculus, the extreme capsule, the inferior longitudinal fasciculus and even the newly discovered frontal aslant tract or FAT (Dick et al., 2014; Dick et al., 2018; Dick and Tremblay, 2012). A more recent avenue of research focuses on how experiences can induce neuroplasticity and affect speech-language skills, including learning new languages, engaging in musical activities or in cognitive training programs. In sum, research on the neurobiology of speech and language in the healthy brain is blossoming.

Importantly, fMRI studies on patients and healthy participants have provided critical evidence demonstrating that language functions (and cognition in general) rely on large-scale brain networks that interact and partially overlap for some functions (Williams et al., 2022). These findings contributed to a shift of interest from the study of functional segregation to functional integration in fMRI studies. For this reason, alongside fMRI analysis methods for mapping regional changes in BOLD signal associated with neural activity, new methods have been developed to measure the characteristics of the functional interaction among brain regions belonging to the same network, and more specifically functional and effective connectivity (for a review, see Friston, 2011) Functional connectivity is defined as temporal coherence among the different neurophysiological events taking place in spatially distant regions of the brain. Effective connectivity refers to the influence that one neural system exerts over another (Friston, 2011). The 2005–2015 decade was characterized by a considerable growth of the number of publications studying functional and effective connectivity in the language network; this trend continues to this day.

In sum, structural and functional MRI approaches have had a major impact on our understanding of the adult speech-language network, its development and aging, capacity for reorganization following brain damage or experience.

### 5.3 Brain stimulation approaches

Multiple techniques have been used to probe the human speech/language system, including direct electrical intracortical stimulation and transcranial magnetic stimulation (TMS). The first intraoperative brain mapping studies using direct electrical stimulation were conducted in the 1950s in Montreal, Canada, by neurosurgeon Wilder Penfield and neurophysiologist Herbert Jasper. By applying electrical currents to the cortical surface of the brain during surgery, Penfield and Jasper mapped motor, sensory, and language functions in awake patients and identified key regions for speech production and comprehension (Jasper and Penfield, 1949). Their research mapped language functions, laying the groundwork for modern techniques like electrocorticography (ECoG) and functional MRI (fMRI). The intracranial stimulation approach is still used today during awake surgery for patients with brain tumor to minimize postoperative disorders. Studies using this method continue to provide insightful contributions to the neurobiology of speech and language (e.g., Ramirez-Ferrer et al., 2024).

Since the emergence of human brain stimulation, less invasive techniques have emerged, including TMS, which can be used on both healthy adults and those with neurological disorders. The principle of electromagnetic induction—which is the core mechanism for TMS—was discovered in the nineteenth century by the English physicist Faraday (1839): a rapidly changing magnetic field can generate an electric current in a nearby conductive element. This important discovery was followed by decades of experimentation. In 1896, the French physicist Jacques-Arsène d’Arsonval observed that when people position their head on a coil emitting a magnetic field, they report visual sensations (George et al., 2007). One of the first attempts to use a technique similar to TMS for clinical purposes took place in 1902, when Australian psychiatrists Adrian Pollacsek and Berthold Beer reported using an electromagnetic coil, positioned above the head to treat depression and neuroses (George et al., 2007). Over 50 years later, American researcher Kolin et al. (1959) were the first to trigger a muscle contraction in the sciatic nerve of a frog following magnetic stimulation of the sciatic nerve. The modern TMS devices appeared in 1985, following the work of English researcher and medical physicist Anthony Barker and his team. It was this team that first documented hand movements induced by TMS and the electrical activity associated with it in humans (Barker et al., 1985).

Nowadays, we know that TMS can inhibit or enhance cortical excitability. This is accomplished via localized magnetic field pulses that vary in intensity, frequency and number. TMS can be used to identify brain regions that participate in speech and language-related processes and clarify their functions. The use of TMS to understand the neurobiology of speech and language originally relied on inducing reversible “virtual lesions,” akin to those produced by the more invasive Wada tests, in cerebral cortical areas involved in language function, using inhibitory protocols. Most studies employing the virtual lesion approach used low frequency repetitive TMS to inhibit functions. In 1991, American researcher Pascual-Leone et al. (1991) and his team published a study in which they applied inhibitory repetitive TMS (rTMS) to regions of the left hemisphere of the brain. rTMS resulted in interrupted speech production, a phenomenon often referred to as a “speech arrest.” Importantly, speech arrests are not typically caused by stimulating the posterior IFG (Broca’s area). Rather, and consistent with knowledge about the cellular architecture and connectivity of the IFG and adjacent premotor cortex, speech arrests are more frequently caused by stimulation to the adjacent premotor cortex. TMS therefore played an important part in understanding that “Broca’s area” is not strictly speaking a motor center for speech. Since then, hundreds of studies have been conducted examining various aspects of human communication through inhibitory rTMS, including speech processing, speech production, phonological working memory, auditory comprehension, naming, reading, pitch regulation and many others.

TMS can be leveraged for therapeutic applications in neurology, psychology, audiology and speech-language pathology, to name but a few domains of application. It can generate facilitation through enhanced cortical excitability as well as inhibit hyperactive systems to restore performance. Initially, therapeutic inquiries with TMS focused heavily on PWA, attempting to enhance language recovery following a stroke. Most TMS studies in PWA have used inhibitory low frequency rTMS applied to the contralesional triangular IFG to reduce right hemisphere hyperactivity and transcallosal inhibition on the left IFG. A meta-analysis involving 160 PWA found a positive, though limited, effect of this approach on language recovery (Ren et al., 2014). More recent studies have employed excitatory TMS approaches. The development of new protocols, such as intermittent theta-burst stimulation (iTBS) (Huang et al., 2005), which is associated with long-term potentialization (Tang et al., 2017), has allowed researchers to enhance various processes including speech processing, auditory comprehension and semantic cognition. The iTBS protocol consists of trains of three rapid pulses, presented at 50 Hz and repeated at a 5 Hz frequency for 2 s, every 10 s (total of 600 pulses in 3 min). Excitatory rTMS such as iTBS is being studied to enhance speech and language functions in people with neurological and neurodegenerative disorders but also older adults.

Importantly, TMS can be combined with brain imaging and neurophysiological techniques. MRI-guided TMS—the norm in research contexts—allows researchers to identify targets on participants’ brain MR images. TMS combined with EEG allows researchers to study the impact of TMS on speech and language networks at rest or during a task. This type of paradigm could help reveal the nature of the important inter-subject variability in the effect of TMS and lead to more personalized approaches.

In sum, TMS is a versatile approach that can be used to study brain-language relationships as well as enhance or restore functions. Now that many brain systems have been thoroughly characterized via brain imaging and neurophysiological methods, the use of TMS to target functions and systems will be easier and more effective.

## 6 Current models

Advances in methodology and interdisciplinary research have led to increasingly sophisticated models of the neurobiology of language. The profusion of neurophysiological, brain imaging, and brain stimulation studies that have been conducted in the past four decades, inquiring into various aspects of language production and comprehension have complemented—and often challenged—classical views on the neurobiology of language. Although no method is flawless, leveraging their complementarity has pushed the research community forward into a word of possibilities. For instance, enhanced knowledge of brain architecture and connectivity has changed our understanding of the role of “Broca’s area.” The IFG (including Broca’s area), is not an agranular (motor) region, and it does not connect to the descending (motor) tracts. As such, it cannot directly control articulation. Broca’s area is therefore no longer seen as a motor speech center. Instead, contemporary models of speech production recognize a major role in articulation and motor control to the primary motor (M1) and adjacent ventral premotor cortex (PMv), which have direct connection to the descending motor pathways, and have either an agranular (M1) or a dysgranular (PMv) architecture.

Another significant advance is the understanding that the lateralization of language is not absolute. Certain functions, such as speech perception and articulation, engage both hemispheres, while others may predominantly rely on the right hemisphere, including prosody, pragmatics, and non-literal aspects of language. Here, we briefly introduce two of the most dominant contemporary models: the dual-stream speech processing model, and the DIVA/GODIVA models.

The dual-stream speech processing model, developed by Hickok and Poeppel in the early 2000s (Hickok, 2009; Hickok and Poeppel, 2004, 2007), is perhaps the most prominent framework for understanding how the brain processes spoken language. The model builds from the classical Wernicke-Lichtheim model, proposing two pathways instead of one: a dorsal stream, which links auditory processing to motor functions for speech production, involving the posterior superior temporal gyrus (STG), the planum temporale, and the inferior parietal lobule, projecting to frontal regions including the IFG; and a ventral stream, which maps speech sounds to meaning, involving the middle and inferior temporal gyri projecting to anterior temporal and frontal regions. Despite its value, the dual-stream model has limitations, including oversimplification and inability to account for individual variability, learning and contextual factors. Ongoing research continues to refine this framework.

Models of speech production have also advanced significantly. While Levelt (1993, 1999) offered a detailed account of the linguistic planning that precedes articulation (Indefrey and Levelt, 1999), their model lacks specificity regarding speech motor control and connections with the domain-general motor system. The Directions into velocities of articulators (DIVA) model (Guenther et al., 1998; Tourville and Guenther, 2010), developed by Guenther (2016), is a model of speech production and speech learning that focuses on feedforward and feedback mechanisms. The more recent Goal-oriented dynamics of articulators (GODIVA) model (Bohland et al., 2010) extends DIVA by incorporating dynamic aspects of articulatory movements, temporal dynamics, and improved error correction. Key brain areas in the GODIVA model include M1 for generating motor commands, the PMv which contains speech sound representations, the IFG for grammatical structures, the basal ganglia for motor control and error correction, and the cerebellum for fine-tuning articulatory movements based on feedback. Importantly, the DIVA and GODIVA models envision speech motor control within the broader scope of motor control.

Despite these enormous advances, models incorporating speech/language processing and speech production mechanisms are still lacking.

## 7 Contemporary challenges

While it began as a medical endeavor, the field of language neurobiology has evolved tremendously to become an intersectoral research field which combines methods and theories from the social sciences and humanities (linguistics, psychology, cognitive sciences), the natural sciences (e.g., neuroscience, biology, genetics, physics, biomedical engineering) and the health sciences (e.g., neurology, speech-language pathology, neuropsychology, genetics, psychiatry and audiology). This interdisciplinarity broadens the scope of inquiries into the neurobiology of language and yields increasingly sophisticated research outcomes, but it also presents challenges, such as adopting common terminology and adjusting cross-domain publishing cultures and approaches.

Current challenges involve developing more advanced models that account for language development from infancy through old age, adapt to changes resulting from trauma, and incorporate the acquisition of new skills, including both linguistic and musical abilities. Integrating models of speech and models of language functions is both essential and challenging to understand communication more holistically. Further, while models of speech production have evolved to increasingly integrate domain-general motor infrastructure, sub-cortical structures are still largely absent from language models. Methodologically, the field would benefit from incorporating more naturalistic stimuli and environments to assess communication in situations akin to real life. Comparing controlled and less controlled stimuli and tasks could yield valuable insights. As in other fields of cognitive neuroscience, there is a need for larger participants samples. This has been addressed in part by big data initiatives that provide access to sizable samples. However, in many such samples, the speech-language characterization is very limited, which constrains the type of questions that can be asked. Further, most datasets are focused on investigating neurological populations, such as Alzheimer disease and PWA. Finally, the study of development, aging and brain plasticity is best studied using longitudinal approaches and randomized-controlled studies, which remain scarce. Such approaches are time-consuming and costly and therefore not widely available to most research teams.

## 8 Conclusion

Given the lack of suitable animal models, the study of the neurobiology of language relies heavily on human research, which comes with inherent limitations such as restricted anatomical precision, challenges in assessing microstructural organization, and massive inter-individual heterogeneity. However, as discussed in this essay, advancements in methods for recording brain activity and modulating cortical excitability have broadened the scope of investigation and accelerated discoveries in the field. While no single method is without limitations, their combined use has substantially advanced our understanding and led to more sophisticated models of the neurobiology of language. Additionally, integrating diverse perspectives and disciplines has enriched research questions and outcomes over time. Fostering interdisciplinary dialog and developing intersectoral initiatives should remain the gold standard in the field to address questions related to the neurobiology of speech and language comprehensively and across the lifespan.
